# Aurora-A Interacts with AP-2α and Down Regulates Its Transcription Activity

**DOI:** 10.1371/journal.pone.0023110

**Published:** 2011-08-01

**Authors:** Lihui Zou, Yimin Sun, Mingrong Wang, Qimin Zhan

**Affiliations:** 1 State Key Laboratory of Molecular Oncology, Cancer Institute and Cancer Hospital, Chinese Academy of Medical Sciences and Peking Union Medical College, Beijing, China; 2 CapitalBio Corporation, Beijing, China; Instituto Nacional de Câncer, Brazil

## Abstract

Aurora-A is a serine/threonine protein kinase and plays an important role in the control of mitotic progression. Dysregulated expression of Aurora-A impairs centrosome separation and maturation, which lead to disrupted cell cycle progression and tumorigenesis. However, the molecular mechanism by which Aurora-A causes cell malignant transformation remains to be further defined. In this report, using transcription factors array and mRNA expression profiling array, we found that overexpression of Aurora-A suppressed transcription activity of AP-2α, a tumor suppressor that is often downregulated in variety of tumors, and inhibited expression of AP-2α-regulated downstream genes. These array-based observations were further confirmed by microwell colorimetric TF assay and luciferase reporter assay. Downregulated transcription activity of AP-2α by Aurora-A was found to be associated with reduced AP-2α protein stability, which appeared to be mediated by Aurora-A enhanced ubiquitin-dependent proteasomal degradation of AP-2α protein. Interestingly, Aurora-A-mediated AP-2α degradation was likely dependent Aurora-A kinase activity since inhibition of Aurora-A kinase activity was able to rescue Aurora-A-induced degradation of AP-2α. Moreover, we defined a physical interaction between Aurora-A and AP-2α, and such interaction might bridge the suppressive effect of Aurora-A on AP-2α protein stability. These findings provide new insights into molecular mechanism by which Aurora-A acts as an oncogenic molecule in tumor occurrence and malignant development.

## Introduction

Aurora-A is an important member of Aurora kinase family that includes Aurora-A, Aurora-B and Aurora-C. This serine/threonine protein kinase family plays critical roles in the control of centrosome separation, bipolar spindle assembly, chromosome segregation and cytokinesis[1], [2], [3]. Human Aurora-A locates on chromosome 20q13, a region that is frequently amplified in primary breast tumors, colorectal cancers, ovarian cancers, neuroblastoma and multiple cancer cell lines[4], [5]. A number of investigations demonstrate that Aurora-A is overexpressed in human esophageal squamous cell carcinoma (ESCC). Overexpression of Aurora-A is associated with poor differentiation and malignant status of clinical ESCC. Levels of Aurora-A protein are correlated to the migrating potentials of tumor cells[6]. In addition, Aurora-A promotes cancer cell proliferation and inhibits cisplatin- or UV-induced apoptosis, probably through its up-regulation of Bcl-2 expression[7]. These observations strongly suggest that Aurora-A is dysregulated in human ESCC and plays an important role in the development of ESCC although further investigations are required for elucidating the underlying mechanism.

Members of AP-2 transcription factor family contain a DNA binding domain at the C-terminus and an activation domain at N-terminus. AP-2α is the first identified AP-2 family member, which binds to a typical consensus sequence (5′-GCCNNNGGC-3′) as homodimers or heterodimers and regulates the gene expression. AP-2α is greatly involved in the control of cell proliferation and differentiation[8], [9], cell cycle progression[10], [11] and apoptosis[12]. Multiple evidence indicate that AP-2α acts as a tumor-suppressor gene. Reduced expression of AP-2α is often detected in several types of cancer, such as breast cancer, cutaneous malignant melanoma, prostatic carcinoma, neuroglioma [13]-[17], indicating that loss of AP-2α function may contribute to tumorigenesis and development of tumor malignancy.

To elucidate the molecular mechanism of Aurora-A in the occurrence and development of ESCC, we attempted to investigate the regulatory roles of Aurora-A in the control of global gene expression. We utilized two high-throughput methodologies: the mRNA expression profiling array and oligonucleotide array-based transcription factors assay to analyze the effect of Aurora-A on gene transcription. We found that in cells expressing high Aurora-A, AP-2α transcription activity was down regulated, which was coupled with downregulation of AP-2α-regulated downstream genes. Furthermore, we observed that Aurora-A was able to interact with AP-2α and decrease AP-2α protein stability by promoting proteasome mediated protein degradation. These findings demonstrate the functional connection between Aurora-A and AP-2α and the suppressing role of Aurora-A in the control of AP-2α-mediated transcription.

## Materials and Methods

### 1. Cell cultures, transfection and treatment

Human ESCC KYSE150 and ESCC EC9706 cell lines, which were generously provided by Dr Shimada in Kyoto University, were grown in RPMI 1640 (Invitrogen) supplemented with 10% fetal bovine serum, 100 U/ml penicillin and 100 µg/ml streptomycin, maintained at 37°C humidified atmosphere of 5% carbon dioxide. pEGFP-Aurora-A transfected KYSE150 cells and Aurora-A siRNA transfected EC9706 cells were cultured in the presence of 400 µg/ml of G418 (Geneticin sulfate, GIBCO). HCT116 p53^+/+^ and HCT116 p53^-/-^ cells (gifts from Dr. Bert Vogelstein, Johns Hopkins University) were grown in DMEM supplemented with 10% FBS. For cell transfection, KYSE150/GFP-Aurora-A and KYSE150/GFP cells, which were stable cell lines through stable transfection of pEGFP-Aurora-A or pEGFP empty vector in human ESCC KYSE150 cells[7], were seeded onto 96-well plates one day prior to transfection. In each well, 0.1 µg of DNA and 0.25 µl of Lipofectamine2000 (Invitrogen) were added to 50 µl of RPMI 1640 (Invitrogen). Fresh medium was added at 6 h after transfection, and cells were harvested 48 h later. For analysis of protein stability, 100 µg/ml CHX (cycloheximide) (Sigma–Aldrich Corp., St. Louis, MO) or 10 µmol/L MG132 (Sigma–Aldrich Corp., St. Louis, MO) was added to cell medium, and then cells were harvested at the indicated time points. The Aurora-A kinase inhibitor III (Merck Calbiochem Inc. Darmstadt, Germany, 189405) dissolved in DMSO and stored at 50 mg/ml at −20°C, was used at 1 µM for 2 h prior to treatment with CHX.

### 2. Gene expression profiling array analyses

Total RNAs from the KYSE150/GFP-Aurora-A and KYSE150/GFP cell lines were extracted using Trizol reagent according to the manufacturer's instructions (Invitrogen). The RNA (5 µg) was used to produce a KIENOW-labeled cDNA probe for hybridization of a expression array, which contain 21,571 probes (CapitalBio Corporation, Beijing, China), and analyzed using a LuxScan10 K/A laser scanner (CapitalBio Corporation, Beijing, China). The results were imported into GenePix Pro4.0 software (Axon instrument company) for data mining. Statistical comparisons were made using Student's t test by the SPSS software. A significant difference was defined as P<0.05[19].

### 3. Single Primer Amplification assisted Oligonucleotide array-based transcription factor assay (SPA-OATFA)

We performed transcription factor assays based on oligonucleotide arrays. Nuclear extracts were prepared by nuclear extraction kit (Active Motif, Carlsbad, CA), the single primer amplification (SPA) and the array hybridization was performed using oligonucleotide array-based transcription factor assay (OATFA) according to the manufacturer's instructions as described previously (CapitalBio Corporation, Beijing, China) [20], [21].

### 4. Nucleoprotein extraction and Western Blot analysis

Cells were harvested, rinsed with PBS and nuclear extracts were prepared by NE-PER nuclear and cytoplasmic extraction Kit (Pierce, Rockford, IL). 100 µg of proteins were used for electrophoresis 10% SDS–PAGE. After electrophoresis, the proteins were transferred to nitrocellulose membranes. Membranes were blocked in 5% milk, washed with PBST (PBS with 0.1% Tween), and incubated with anti-Aurora-A (Cell Signaling, Beverly, MA, 3092), AP-2α (Santa Cruz Biotechnology, Santa Cruz, CA, sc-12726), and actin antibodies (Santa Cruz Biotechnology, Santa Cruz, CA, sc-8532). Following washing and incubation with horseradish peroxidase-conjugated anti-rabbit or anti-mouse antibody at 1∶3000 in 5% milk, the membranes were washed and detected by ECL (Applygen Technologies Inc., Beijing, China) and exposure to x-ray film[22].

### 5. Microwell colorimetric TF assay (ELISA)

Microwell colorimetric TF assay was performed as reported by Renard et al[23]. Nuclear proteins from the KYSE150/GFP-Aurora-A and KYSE150/GFP cell lines were incubated with AP-2α binding DNA double-stranded oligonucleotidic probe on multi-well plates and the AP-2α antibody was added. Subsequently, the peroxidase-conjugated anti-mouse IgG was bound to the anti-AP-2α antibodies. After coloration for 15 min, the absorbance of each well was measured at 450 nm with an ultramark microplate reader (Bio-Rad, Hercules, CA). Lysis buffer was incubated in the microwells instead of nuclear proteins as blank, and the results were calculated after subtraction of the blank values.

### 6. Luciferase assays

The (AP-2)_3_-TK-LUC expression vector was a gift from Dr Carlo M. Croce of Ohio State University[24]. Three repeats of the AP-2–binding site were cloned upstream of the basic promoter of thymidine kinase (TK) in the pGL3 (Promega, Madison, WI). KYSE150/GFP-Aurora-A and KYSE150/GFP cells were transiently transfected with AP2-LUC. To normalize for transfection efficiency, cells were cotransfected with pRL- SV40 expression vector (Luciferase Assay System, Promega). All transfection assays contained 100 ng of reporter plasmid and 1.0 ng of pRL-SV40 plasmid. Meanwhile, the cells were cotransfected with empty pGL3-Basic vector and pRL-SV40 as controls. Cells were collected 48 hours later, rinsed with PBS, resuspended in reporter cell lysis buffer (Promega, Madison, WI), and incubated for 15 minutes at room temperature. Insoluble material was spun down, and luciferase activity was quantified according to the manufacturer's protocol of the luciferase assay kit (Promega, Madison, WI). Results were shown as fold induction of the luciferase activity read by the manual luminometer (TD-20/20*^n^*; Turner Designs, Sunnyvale, CA) compared with control cells. Differences were determined by *t*-test, and *P*<0.05 was considered significant.

### 7. Immunoprecipitation

For immunoprecipitation, 1 mg of cellular protein was first incubated with 7.5 µg of the indicated anti-Aurora-A, AP-2α antibodies for 6 h at 4°C and further incubated overnight at 4°C after addition of 10 µl of protein A/G-agarose beads (Santa Cruz Biotechnology, Santa Cruz, CA). Immunoprecipitates were washed six times with lysis buffer and loaded onto SDS-PAGE gel and transferred to nitrocellulose membranes for immunoblotting analysis[25].

### 8. GST pull-down assays

GST pull-down assays were performed with recombinant Aurora-A-GST protein and GST protein alone expressed in E. coli. GST fusion protein and GST protein were bound to 15 µl glutathione-sepharose beads for 6 h at 4°C. The sepharose beads were then incubated with a mixture of protein extraction for 6 h at 4°C with gentle rotation. After being washed with lysis buffer for 3–5 times gently, the beads incubated were boiled in SDS–PAGE loading buffer and analyzed by Western Blot assay.

## Results

### 1. Transcription activity of AP-2α is downregulated by Aurora-A

**Figure 1:**
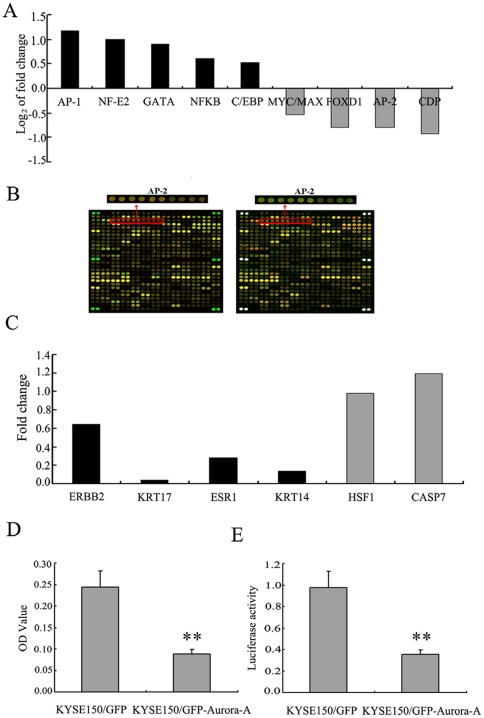
Transcription activity of AP-2α is downregulated by Aurora-A. (A) The summary of the transcription factors with significant changes in their activities by transcription factors array when Aurora-A was overexpressed. (B) Fluorescence images of the transcription factors arrays hybridized with nuclear extracts from KYSE150/GFP-Aurora-A and KYSE150/GFP cells. The analysis of arrays was replicated twice, exchanging the Cy3- and Cy5- label. Magnified panels show the spots representing the activity of AP-2 transcription factor that was down-regulated upon Aurora-A overexpression. (C) The summary of the AP-2α-regulated genes with significant down-expression when Aurora-A was overexpressed by mRNA expression profiling array. The black bars represent the AP-2α-regulated genes, and the gray bars represent the genes which were not the AP-2α-regulated genes. (D) The nucleoprotein extraction of KYSE150/GFP-Aurora-A and KYSE150/GFP cells were incubated with AP-2α binding DNA double-stranded oligonucleotidic probe on multi-well plates, then anti-AP-2α antibodies and the peroxidase-conjugated anti-mouse IgG were subsequently added. At last, the samples could be read for the colorimetric detection. (E) KYSE150/GFP-Aurora-A and KYSE150/GFP cells were cotransfected with (AP-2)_3_-TK-LUC expression vector and a pRL-SV40 expression vector. Transcription activities were expressed as luciferase values after normalization. All experiments were performed three times and described as mean ± SD.

In spite of the understanding on the roles of Aurora-A in the mitotic progression, little is know about its effect on transcriptional regulation. Therefore,the single primer amplification assisted oligonucleotide array-based transcription factor assay (SPA-OATFA) was employed to characterize transcriptional factors that might be regulated by Aurora-A. Using such TF array that contains total 240 transcription factors, we first compared activities of global transcription factors in both cells expressing high levels of Aurora-A (KYSE150/GFP-Aurora-A) and control cell line (KYSE150/GFP). As shown in Figure 1A and 1B, 5 transcription factors (AP-1, NF-E2, GATA, NFKB, C/EBP) exhibited up-regulated transcription activities, but 4 transcription factors (MYC/MAX, FOXD1, AP-2 and CDP) showed significant downregulation of their transcription activity in KYSE150/GFP-Aurora-A cells. These data suggest that overexpression of Aurora-A might affect activities of a group of important transcription factors. Since AP-2α (TFAP2A) is often downregulated in human cancers and acts as a tumor-suppressor gene [14]–[17], the further efforts were especially made in understanding how AP-2α is regulated by Aurora-A.

Meanwhile, we carried out analysis of mRNA expression profiling to search for the genes regulated by Aurora-A. This mRNA expression array included 21,571 genes of human genome. Totally, 209 differentially expressed genes were identified in KYSE150/GFP-Aurora-A compared to KYSE150/GFP cells, including 129 up-regulated genes (cut off >2.0) and 80 down-regulated genes (cut off <0.5). In support of the observations that Aurora-A suppresses AP-2α activity, we found that a group of AP-2α-regulated genes, including ERBB2[26], KRT17[27], ESR1[28], [29], KRT14[30]were significantly downregulated when cells express high level of Aurora-A (Figure 1C). HSF1 and CASP7 were not the AP-2α-regulated genes, and the expression was not affected by Aurora-A overexpression.

Usually, using SPA-OATFA as the first stage screening tool, researchers can rapidly select potential candidate. However, further antibody-based methods should be performed to validate the SPA-OATFA data. We first utilized the microwell colorimetric TF assay to verify the SPA-OATFA results. Following binding of the double-stranded oligonucleotidic probes on multi-well plates, nuclear extracts were added to the microwells and incubation with mouse anti-AP-2α antibodies. Subsequently the peroxidase-conjugated anti-mouse IgG was bound to the anti- AP-2α antibodies. Finally, the absorbance of each well was measured, which reflects AP-2α protein activity. Consistent with the observations in SPA-OATFA assay, AP-2α activity was obviously downregulated in the KYSE150/GFP-Aurora-A compared to the KYSE150/GFP (Figure 1D).

We also employed the reporter gene assay to determine the influence of Aurora-A on AP-2α activity. The KYSE150/GFP-Aurora-A and KYSE150/GFP cells were transiently transfected with *AP2-LUC* plasmid, a luciferase reporter cloned downstream of TFAP2-binding elements. To normalize the transfection efficiency, cells were cotransfected with pRL-SV40 expression vector. Luciferase activity was quantified using a luminometer. Results were shown as fold induction of the luciferase activity. Clearly, AP-2α activity was downregulated in the KYSE150/GFP-Aurora-A cells compared to the KYSE150/GFP (Figure 1E).

### 2. Aurora-A downregulates AP-2α protein expression in a p53-independent manner

**Figure 2:**
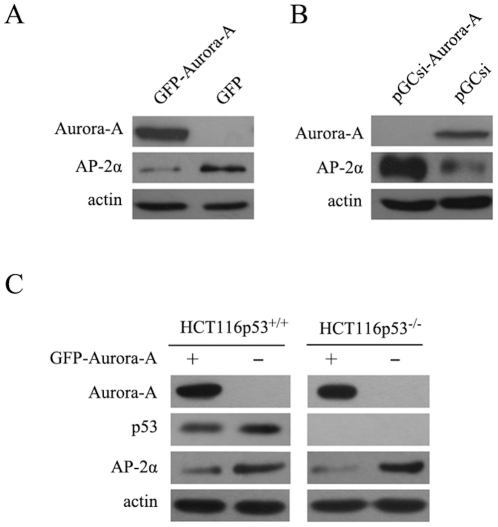
Regulation of AP-2α gene expression by Aurora-A. (A) AP-2α protein levels in KYSE150/GFP-Aurora-A and KYSE150/GFP cells were analyzed using immunoblotting assay. The actin levels were measured as loading control. (B) AP-2α protein levels in EC9706/pGCsi???EC9706/pGCsi-Aurora-A cells were examined by Western Blotting assay. (C) The HCT116 p53^+/+^ and HCT116 p53^-/-^ cells were transiently transfected with pEGFP-Aurora-A and control plasmids. Cells were lysed after 48h of incubation and evaluated for the expression of AP-2α by Western Blotting assay.

It is well accepted that activity of transcription factors is closely related to their protein levels and protein modifications. We further examined the effect of Aurora-A on the protein level of AP-2α. Total cellular proteins were prepared from KYSE150/GFP-Aurora-A and KYSE150/GFP cells and subjected to immunoblotting assay with AP-2α antibody. As shown in Figure 2A, AP-2α protein levels were much lower in KYSE150/GFP-Aurora-A than that seen in KYSE150/GFP cells. Additionally, siRNA approach was used to confirm suppressive effect of Aurora-A on AP-2α. Following knockdown of endogenous of Aurora-A in EC9706/pGCsi-Aurora-A cells, AP-2α protein expression was clearly up regulated in contrast to the control cells (EC9706/pGCsi) (Figure 2B). These results indicate that Aurora-A is able to affect AP-2α protein level, which contributes to downregulation of AP-2α transcription activity.

Previous studies have demonstrated that phosphorylation by Aurora-A induces MDM2-mediated destabilization and inhibition of tumor suppressor p53, and in turn results in abrogation of p53 DNA binding and transactivation[31], [32]. In addition, Li et al have reported that AP-2α is a transcriptional target of p53[33]. So the question raised here is whether Aurora-A downregulation of AP-2α is correlated to Aurora-A inhibition on p53. To confirm this hypothesis, HCT116 p53^+/+^ and HCT116 p53^-/-^ cells (a somatic knock-out for p53 derived from HCT116 cell line, gifts from Dr. Bert Vogelstein, Johns Hopkins University) were transfected with pEGFP-Aurora-A and control plasmids. Following 48h of incubation, we collected cellular proteins and examined the levels of AP-2α in both cell lines. While, the downregulation of AP-2α protein level was seen in both cell lines after introduction of Aurora-A expression vector into cells (Figure 2C). Likely, Aurora-A downregulates AP-2α protein level in a p53-independent manner.

### 3. Overexpression of Aurora-A enhances the proteasome-dependent degradation of AP-2α

**Figure 3:**
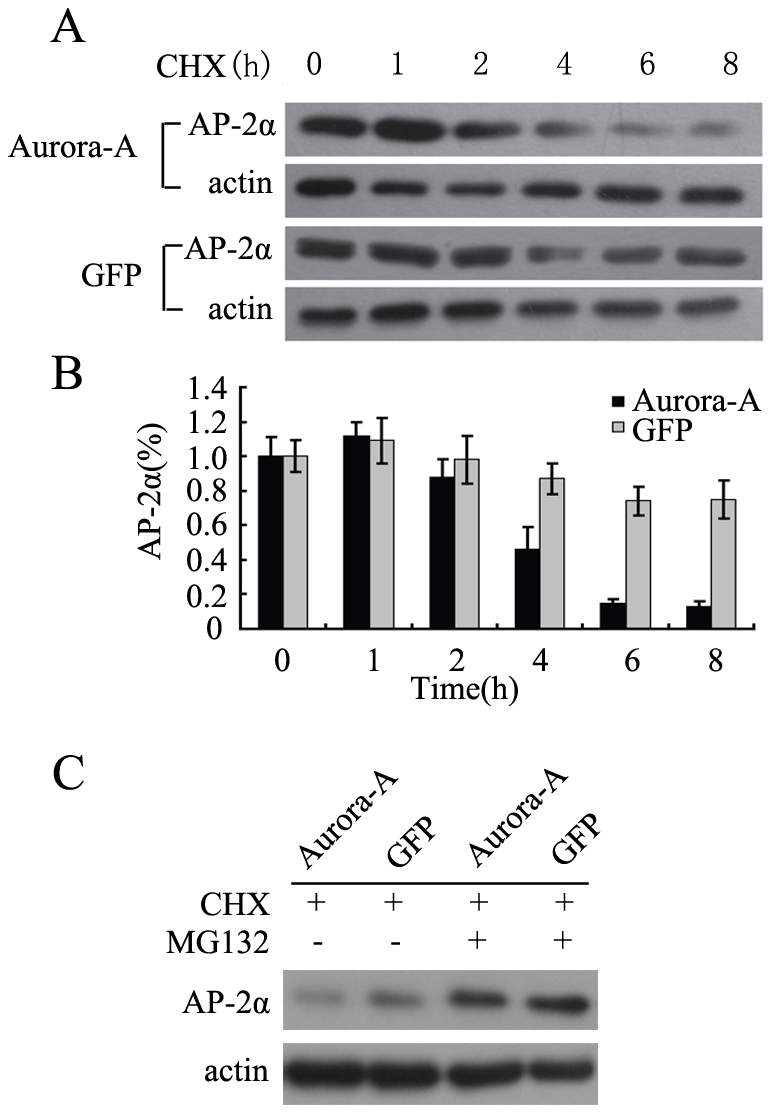
Overexpression of Aurora-A enhances the degradation of AP-2α. (A) KYSE150/GFP-Aurora-A and KYSE150/GFP cells were treated with CHX respectively, and then harvested at the indicated time points. AP-2α protein level at each time point was determined by Western Blot. (B) The amounts of AP-2α were calculated by densitometry and normalized to corresponding actin levels. The column diagram represents the amount of normalized AP-2α at each time point comparing with the original levels (0 h). (C) Aurora-A enhances the proteasome-dependent degradation of AP-2α. KYSE150/GFP-Aurora-A and KYSE150/GFP cells were treated with CHX alone or combination with MG-132. AP-2α protein level was determined by Western Blot.

Next, we further investigated biochemical mechanism(s) by which Aurora-A downregulates AP-2α protein. We examined the stability of AP-2α protein in both KYSE150/GFP-Aurora-A and KYSE150/GFP cell lines treated with protein synthesis inhibitor cycloheximide (CHX) for different time points (0, 1, 2, 4, 6 and 8 h). As shown in Figure 3A and 3B, in KYSE150/GFP cells, AP-2α protein was stable with a long half-life, and protein level was decreased only 20% after 8 h. In contrast, in Aurora-A overexpression cells, the level of AP-2α was reduced more than 50% at 4 h after treatment with CHX, and its level was less than 20% after 6 h. These results suggest that Aurora-A overexpression enhances the degradation of AP-2α.

It has been reported that the proteasomal-degradation pathway controls the stability of AP-2α protein[34]. It is reasonable to suspect that Aurora-A enhanced degradation of AP-2α might be associated with the proteasomal-degradation pathway. Thus, both KYSE150/GFP-Aurora-A and KYSE150/GFP cells were treated with CHX alone or treated with CHX in the presence of MG132, a selective inhibitor of the proteasome and followed by the analysis of AP-2α protein levels via immunoblotting analysis. As shown in Figure 3C, AP-2α protein was clearly accumulated in both cell lines following treatment with MG132. These data suggest that the effect of Aurora-A on AP-2α stability might be mediated through activation of proteasome-dependent pathway.

### 4. Aurora-A enhanced AP-2α degradation depends on its kinase activity

**Figure 4:**
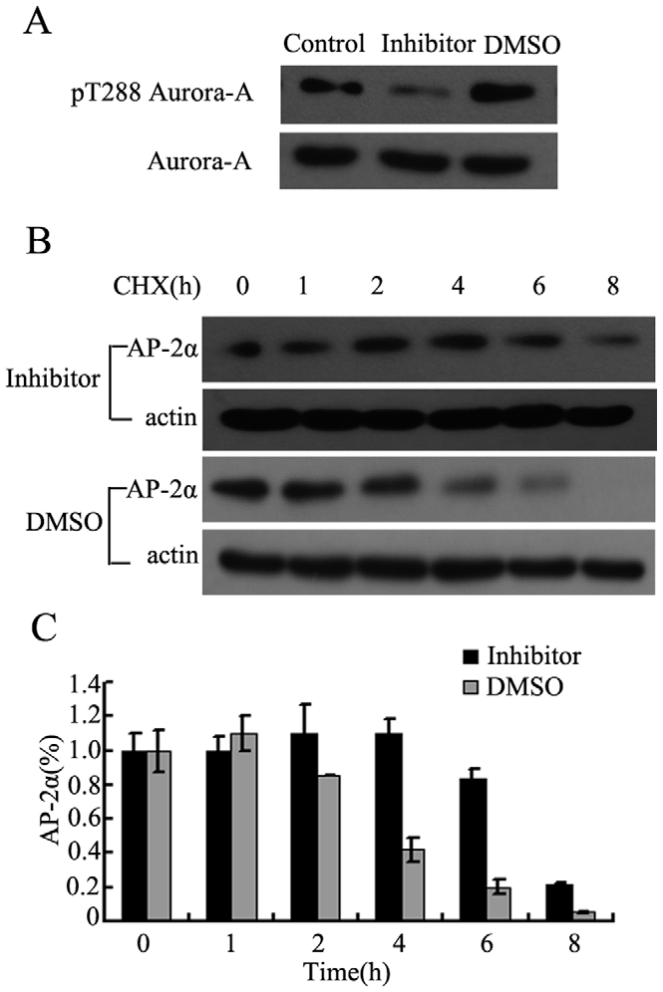
Aurora-A-mediated AP-2α degradation depends on Aurora-A kinase activity. (A) KYSE150/GFP-Aurora-A cells were exposed to 1 µM of Aurora-A kinase inhibitor and cellular proteins were collected 2 hours later. Western Blot was performed with antibodies to pThr288 on Aurora-A or total Aurora-A. DMSO was used as a negative control. (B) KYSE150/GFP-Aurora-A cells were exposed to Aurora-A kinase inhibitor or DMSO for 2 hours prior to treatment with CHX. Cells were harvested at the indicated time points and analyzed by Western Blot. (C) The amounts of AP-2α were calculated by densitometry and normalized to corresponding actin levels. The column diagram represents the amount of normalized AP-2α at each time point comparing with the original levels (0 h).

To investigate whether Aurora-A-induced AP-2α degradation relies on Aurora-A kinase activity, KYSE150/GFP-Aurora-A cells were treated with a specific inhibitor (Aurora-A kinase inhibitor III). It has been demonstrated that Aurora-A kinase activity is mainly dependent on its autophosphorylation of Thr288 in the activation loop[35]. Thus, detection of Aurora-A autophosphorylation on Thr288 normally reflects its kinase activity. After KYSE150/GFP-Aurora-A cells exposure to kinase inhibitor for 2 h, reduced Aurora-A autophosphorylation on Thr288 (p-Aurora-A) was evidently detected (Figure 4A). Interestingly, treatment with Aurora-A kinase inhibitor in KYSE150/GFP-Aurora-A cells substantially rescued Aurora-A enhanced AP-2α degradation. In the cells treated with CHX and DMSO, significant reduction of AP-2α protein was seen at two hours, and AP-2α remained only 20% at 6 h after treatment. However, the cells treated with CHX and the Aurora-A kinase inhibitor III exhibited no reduction of AP-2α at 2 or 4 h, and remained 80% of AP-2α protein levels at 6 h posttreatment(Figure 4B, 4C). These observations indicate that Aurora-A kinase activity is required for its role in regulating AP-2α degradation.

### 5. Aurora-A physically interacts with AP-2α

**Figure 5:**
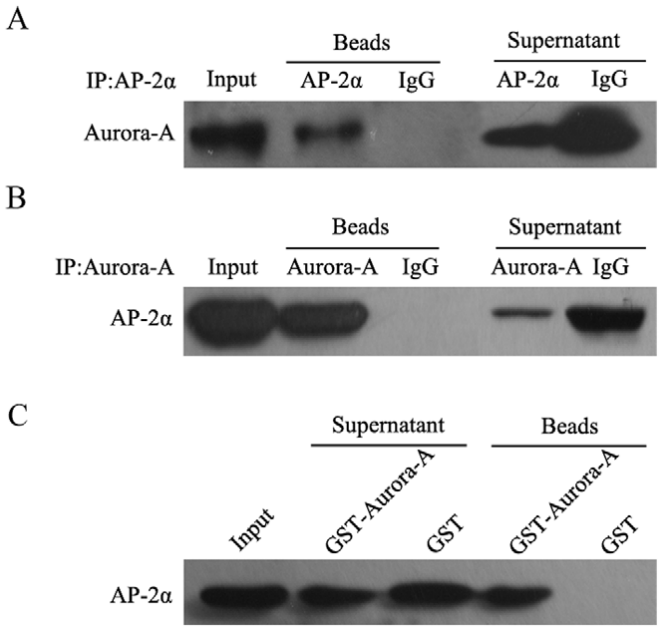
Physical association of Aurora-A with AP-2α. (A‵B) Protein extracts from KYSE150/GFP-Aurora-A cells were prepared and immunoprecipitated with anti-Aurora-A, anti-AP-2α, mouse or rabbit IgG. The immunocomplexes were analyzed by SDS-PAGE and immunoblotted with antibodies against Aurora-A and AP-2α respectively. The visualized bands were shown for Aurora-A and AP-2α. (C) Aurora-A-GST fusion protein was bound to glutathione-sepharose beads, and incubated with protein extracts. The pull-down complexes were analyzed by SDS-PAGE and immunoblotted with antibodies against AP-2α. The visualized band of 48 kDa was AP-2α.

Aurora-A has been shown to interact with multiple cellular proteins in order to regulate their biological functions[31], [32], [36], [37]. Thus, we examined if enhanced AP-2α degradation by Aurora-A might involve the physical interaction between Aurora-A and AP-2α. To address this issue, protein extracts isolated from KYSE150/GFP-Aurora-A cells were incubated with anti-Aurora-A and anti-AP-2α antibodies respectively, and then immunoprecipitated with protein A/G-agarose beads. The immuno-complexes were analyzed by Western Blotting assay, and the results were shown in Fig 5. Aurora-A protein was presented in the immunocomplexes precipitated by the antibodies against AP-2α (Figure 5A), and also AP-2α protein was detected in the immuno-complexes precipitated by the antibodies against Aurora-A (Figure 5B). In contrast, neither Aurora-A nor AP-2α was detected in the precipitates with IgG antibody (control), suggesting a physical interaction of Aurora-A with AP-2α. To further verify Aurora-A interaction with AP-2α, the Aurora-A–GST fusion protein and GST protein alone were incubated with cell lysates. Similarly, AP-2α protein was seen in the pull-down complex by Aurora-A–GST, but not in the complex by GST protein (Figure 5C). Collectively, these results indicate that there is a physical interaction between Aurora-A and AP-2α.

## Discussion

Both of expression level and the kinase activity of Aurora kinases are found to be up-regulated in several human cancers, which are often companied by Aurora-A gene amplification[6], [38]. Overexpression of Aurora-A promotes cancer cell proliferation and xenograph tumor growth in nude mice[4], [34]. The activity of Aurora kinases is finely regulated, mainly by phosphorylation and degradation. Aurora-A interacts with several tumor suppressor proteins, including p53[31], [32], BRCA1[37], Gadd45α[18]. Although Aurora-A exhibits strong oncogenic property, the molecular mechanism of how Aurora-A kinase plays a role in tumorigenesis remains to be further defined.

In our previous studies, we have demonstrated that Aurora-A promotes cell proliferation, invasion and metastasis and antagonizes apoptosis in ESCC cells[7]. To better understand how Aurora-A functions as an oncogenic protein in the control of important cellular processes, we carried out a series of experiments to investigate the effect of Aurora-A on transcription regulation. Through the expression profiling array and single primer amplification assisted oligonucleotide array-based transcription factors assay (SPA-OATFA), we found that a group of transcription factors are possibly regulated by Aurora-A (Figure 1A). Among these downregulated transcription factors, AP-2α was of interest due to its tumor-suppressive function and frequent downregulation in human cancers [14]–[17] and attracted our attention for further detailed studies. Normally, activities of transcription factors often include DNA-binding activity and transcriptional activity. SPA-OATFA is a method for only examining DNA-binding activity in vitro. Thus, in addition to utilizing the microwell colorimetric TF assay to further verify the SPA-OATFA results (Figure 1D), we also performed AP-2α reporter gene assay to demonstrate that the transcriptional activity of AP-2α was also down-regulated by Aurora-A (Figure 1E).

With regard to the mechanism by which Aurora-A suppresses AP-2α transcription activity, it is demonstrated that Aurora-A affects AP-2α proteins stability. In the analysis of AP-2α protein level, we found that there was a great reduction of AP-2α protein in cells expressing high level of Aurora-A (KYSE150/GFP-Aurora-A cell line) compared with the control cells (KYSE150/GFP cell line) (Figure 2A). These observations were further confirmed in Aurora-A knockdown cells (EC9706/pGCsi-Aurora-A), where endogenous Aurora-A was suppressed by Aurora-A siRNA (Figure 2B). Interestingly, we demonstrated that overexpression of Aurora-A increased ubiquitin-dependent proteasomal degradation of AP-2α protein, and treatment of cells with the MG-132 greatly rescued the level of AP-2α protein in the presence of overexpressed Aurora-A. Clearly, downregulations of AP-2α transcription activity and protein stability by Aurora-A are associated with Aurora-A kinase activity. Employment of a specific kinase inhibitor of Aurora-A substantially showed a great rescue of protein degradation of AP-2α by Aurora-A (Figure 4).

It has previously been reported that Aurora-A induced p53 degradation by phosphorylation of Ser-315[31] and that DNA binding and transactivation activity of p53 were abrogated by phosphorylation of Aurora-A[32]. Moreover, Li et al have reported that the tumor suppressor activity of AP-2α is mediated through a direct interaction with p53 and that AP-2α protein may mediate some of the downstream effects of p53 expression[33]. However, when introducing Aurora-A expression vectors into HCT116 p53^+/+^ and HCT116 p53^-/-^ cells, we did not see differential expression levels of AP-2α in these two cell lines with different p53 status, and both cell lines exhibited similar reduction of AP-2α protein (Figure 2C). These finding excludes the involvement of p53 in Aurora-A-downregulation of AP-2α protein level and transcription activity.

There are two main classes of transcription factors: one class is “constitutively active TFs” which are programmed to enter the nucleus to take part in transcription after they are synthesized; the another class is “regulatory TFs”, whose localization and activities are regulated by specific activation signals or modifications[39]. AP-2α normally localizes in the nuclues and exerts its transcriptional activation. We indeed performed immunofluorecent assay to determine whether Aurora-A affects AP-2α subcellular localization and influences its regulation of the downstream target genes. Surprisingly, AP-2α predominantly localized in nucleus in both KYSE150/GFP-Aurora-A and KYSE150/GFP cells (data not show). Thus it appears that overexpression of Aurora-A may not affect AP-2α subcellular localization.

Aurora-A often exerts important functions via its physical and functional interactions with many critical cellular proteins, such as p53[31], [32], cyclin-B1 [36], BRCA1[37], c-Myc[40]. Such interactions lead to altered stability or function of those Aurora-A-associating proteins. Similarly, using Co-IP and GST-pull-down assays, we defined that there was an interaction between Aurora-A and AP-2α, and such Aurora-A interaction with AP-2α might contributes to Aurora-A-mediated AP-2α degradation.

Currently, the underlying machinery involved in Aurora-A-enhanced AP-2α degradation still remains to be further investigated. We suspect that Aurora-A may interfere with the ubiquitin-mediated degradation of AP-2α in different ways. First of all, Aurora-A overexpression might induce phosphorylation and activation of some ligases in ubiquitin system, and then lead to enhanced AP-2α degradation. Second, the physical interaction of Aurora-A with AP-2α may alter the conformation of AP-2α, which could facilitate the ligase and other factors to recognize and bind to AP-2α. Third, Aurora-A could directly phosphorylates AP-2α and then decreases its stability. Although a protein kinase A-mediated phosphorylation at position 239 of AP-2α did not change the stability of AP-2α protein[34], [41], there are some other phosphorylation sites need to be defined in the future and phosphorylation of these sites may influence the stability of AP-2α protein.

Over the past decade, Aurora-A has attracted great attentions in the field of cancer biology due to its potent oncogenic property. However, most of efforts have been focused on exploring its roles in the control of cell cycle progression. Dysregulated expression of Aurora abrogates centrosome maturation, chromosomal segregation and leads to genomic instability[42]–[44]. Thus, the findings that Aurora-A downregulates AP-2α transcription activity and protein expression provide new insights into understanding on Aurora-A-mediated cell transformation and carcinogenesis.
